# Improved dark blood imaging of the heart using radial balanced steady-state free precession

**DOI:** 10.1186/s12968-016-0293-7

**Published:** 2016-10-19

**Authors:** Robert R. Edelman, Marcos Botelho, Amit Pursnani, Shivraman Giri, Ioannis Koktzoglou

**Affiliations:** 1 Department of Radiology, NorthShore University HealthSystem, 2650 Ridge Avenue, Evanston, IL 60201 USA; 2 Feinberg School of Medicine, Northwestern University, Chicago, USA; 3 The University of Chicago Pritzker School of Medicine, Chicago, USA; 4 Siemens Medical Solutions USA, Inc., Chicago, USA

**Keywords:** Radial balanced steady-state free precession, Fast spin-echo, Dark blood imaging, Breath-holding, Cardiovascular magnetic resonance

## Abstract

**Background:**

Dark blood imaging of the heart is conventionally performed using a breath-hold, dual-inversion Cartesian fast spin-echo pulse sequence. Our aim was to develop a faster, more flexible approach that would be less motion-sensitive and provide better image quality. For this purpose, we implemented a prototype radial balanced steady-state free precession (bSSFP) pulse sequence.

**Methods:**

The study was approved by the institutional review board. Six healthy volunteers and 27 subjects undergoing clinically-indicated cardiovascular magnetic resonance (CMR) were imaged using dark blood Cartesian fast spin-echo and radial bSSFP. For patient studies, overall image quality, fat suppression and blood nulling were scored on a 5-point Likert scale. The quality of visualization of the right and left ventricular free walls and septum were individually scored. Streaking and ghosting artifacts were noted, as well as signal dropout in the free wall of the left ventricle.

**Results:**

In volunteer studies, radial bSSFP showed less degradation by cardiac or respiratory motion than fast spin-echo as indicated by visual analysis and calculation of the temporal signal-to-noise ratio. The least motion sensitivity and maximal imaging efficiency were achieved with a single-shot radial bSSFP acquisition using only 35 views (temporal resolution = 95 ms). In patient studies, radial bSSFP images showed fewer motion artifacts and were judged to provide better myocardial visibility, including depiction of the right ventricular free wall, than fast spin-echo.

**Conclusions:**

Dual-inversion radial bSSFP provides the benefits of diminished sensitivity to image artifacts from respiratory or cardiac motion, better myocardial visibility, and improved imaging efficiency compared with standard-of-care Cartesian fast spin-echo for dark blood imaging of the heart.

## Background

Aside from the recent introduction of T1- and T2-mapping [1, 2], cardiovascular magnetic resonance (CMR) techniques in routine clinical use [3] have changed little over the last decade. Pre-contrast imaging with a standard clinical protocol involves the acquisition of cine balanced steady-state free precession (bSSFP), cine phase contrast, and dark blood images. Dark blood images are conventionally acquired using T1- and T2-weighted Cartesian dual-inversion fast spin-echo pulse sequences [4]. A contrast agent is then infused followed by additional imaging.

In our clinical practice, we have observed that the standard-of-care Cartesian dark blood fast spin-echo (FSE) technique is prone to inconsistent image quality, substantially impairing the diagnostic utility of the images. In particular, cardiac and respiratory motion artifacts occur frequently, which is a consequence of the fact that the excited spins must remain within the slice for the whole duration of the fast spin-echo readout (tens of milliseconds) to be properly refocused [5]. Moreover, the relatively long inter-echo spacing of the low-bandwidth, standard-of-care fast spin-echo pulse sequence limits the number of views that can be acquired in each shot and makes the technique inefficient. Consequently, only one or two slices are usually acquired per breath-hold. One can attempt to accelerate the scan using a higher parallel acceleration factor or reduced number of phase-encoding steps, but this comes respectively at the expense of a worsened signal-to-noise ratio (SNR) or blurring along the phase-encoding direction.

With respect to the choice of k-space trajectory, radial imaging techniques are intrinsically less sensitive to motion artifacts than Cartesian ones [6]. For coronary MR angiography, we previously demonstrated that radial quiescent-interval slice-selective (QISS) and radial bSSFP were less sensitive to cardiac and respiratory motion than the corresponding Cartesian approaches [7]. Another benefit of radial scans is that they can be readily accelerated by reducing the number of views without significantly compromising spatial resolution, so long as radial streak artifacts from undersampling are adequately suppressed [8]. We therefore hypothesized that dark blood imaging with radial bSSFP might offer two benefits compared with standard-of-care Cartesian fast spin-echo: (1) improved image quality due to reduced sensitivity to cardiac and respiratory motion, and (2) improved scan efficiency due to the ability to use high undersampling factors and thus fewer shots. In order to test this hypothesis, dark blood Cartesian fast spin-echo and radial bSSFP were compared in six healthy volunteers. In addition, we performed a retrospective analysis of a series of patients who had undergone CMR including both dark blood radial bSSFP and standard-of-care Cartesian fast spin-echo for routine clinical indications.

## Methods

Imaging was performed using a six-element cardiac phased array coil at 1.5 Tesla (Magnetom Avanto, Siemens Healthcare, Erlangen, Germany) with peak gradients and slew rates of 45 mT/m and 200 T/m/s.

### Volunteer study

Six healthy subjects (age 23–53, three female) were imaged using dark blood Cartesian fast spin-echo and radial bSSFP during both breath-holding and free breathing. The purpose of the free-breathing scans was to test the sensitivity of the various pulse sequences to respiratory motion artifacts. The dark blood radial bSSFP imaging technique was modified from a pulse sequence that we originally developed for coronary MR angiography [7]. Notable features of that technique included use of a high radial undersampling factor, small field-of-view, and chemical shift-selective fat suppression. In some cases, saturation bands (20–50 mm thick) were placed over the anterior and posterior soft tissues to help reduce streak artifacts arising from bright fat in the near field of the body array coil. Sequence comparisons included: (1) standard-of-care fast spin-echo. Imaging parameters consisted of an 8-shot acquisition, readout bandwidth = 305 Hz/pixel, TE = 18 ms, echo spacing = 5.8 ms; (2) fast spin-echo, 5-shots, readout bandwidth = 977 Hz/pixel, decreased motion sensitivity option, echo spacing = 3.0 ms; (3) radial bSSFP, 4-shots, 140 views; (4) radial bSSFP, 1-shot, 72 views; (5) radial bSSFP, 1-shot, 35 views. All radial bSSFP scans used readout bandwidth = 1002 Hz/pixel, TE = 1.4 ms, echo spacing = 2.7 ms, with equidistant azimuthal view angles on the order of 15° [9]. For fast spin-echo, a dummy RF excitation was included by default to ensure that spins were in a steady-state prior to readout. The dummy pulse was not included with the radial bSSFP technique as it was not found to improve image quality. For both fast spin-echo and radial bSSFP, a dual-inversion preparation was used to suppress blood pool signal [10, 11]. The acquisition window was fixed at about 650 ms with no attempt to optimize the time delay following the dual-inversion preparation so as to maximize the nulling of the blood pool signal.

Both breath-hold and free-breathing scans were acquired for a single mid-ventricular short-axis slice using multiple measurements. For the breath-hold scans, the contrast-to-noise ratio (CNR) between the left ventricular myocardium and the blood pool was measured using the dual acquisition subtraction procedure, which was applied between the final two images acquired in the breath hold. For this analysis, regions of interest (ROIs) were placed in the left ventricular myocardium and the blood pool (median sizes of 38 and 108 mm^2^, respectively). CNR was defined as: √2*(S_M_-S_B_)/σ_D_, where S_M_, S_B_ are the mean signals in the myocardium and blood pool, and σ_D_ is the standard deviation in the myocardial ROI in the difference image. For free-breathing scans, the temporal SNR (tSNR) was computed as tSNR = μ_M_/σ_M_ where μ_M_ and σ_M_ are the mean and standard deviations in the left ventricular ROI across the multiple images acquired during free breathing. The sharpness of the left ventricular endocardial-blood pool border was computed for both the breath-hold and free-breathing acquisitions based on computer-aided analysis of 60 radial spokes emanating from the center of the left ventricular chamber and passing through perpendicular to the myocardium. Sharpness was computed for each spoke as the inverse of the distance between the 20^th^ and 80^th^ percentile locations in the rise of signal from the dark left ventricular blood pool to the myocardium. To suppress outliers and the unwanted influence of papillary structures with irregular contours, sharpness values were sorted into ascending order and the sharpness value at the 75^th^ percentile was selected.

Statistical analysis of CNR, tSNR and sharpness data were performed using repeated-measures analysis of variance carried out in SPSS software (version 17.0, SPSS Inc., Chicago IL). If analysis of variance testing reached statistical significance (i.e., *P* < 0.05), least significant difference post-hoc tests were performed to identify differences between pairs of protocols.

### Patient study

In addition to the volunteer scans, we performed a retrospective review of clinical MRI studies in which a dark blood radial bSSFP scan (4-shots, 140 views) was acquired in addition to the standard-of-care dark blood fast spin-echo scan during a single scan session within the prior 6 months. A total of 27 patients (16 male, 11 female, age range 15–88 years) were included. Clinical indications were varied, including evaluation of suspected ischemic and non-ischemic cardiomyopathy, cardiac valve disease, and abnormality of the great vessels. For clinical studies, dark blood imaging was performed in either or both short-axis and four-chamber orientations. Imaging parameters are summarized in Table 1. T1-weighted scans were triggered to every R-wave, whereas T2-weighted scans were triggered to every second R-wave. The STIR fast spin-echo sequence was acquired using the default echo time (TE) of 47 ms. For T2-weighted radial bSSFP, a T2-prep time of 60 ms was generally used. However, in some cases, additional images were acquired using a T2-prep time of 90 ms to better highlight a suspected T2-signal abnormality. Dual-inversion radial bSSFP and T1-weighted fast spin-echo acquisitions used chemical shift-selective fat suppression, whereas fat suppression with T2-weighted fast spin-echo was performed using short tau inversion recovery (STIR).

**Table 1:** Imaging parameters for patient studies

Pulse sequence	Radial bSSFP	Cartesian fast spin-echo
Spatial resolution	6 mm × 1.4–1.9 mm × 1.4–1.9 mm	8 mm × 1.4 mm × 1.4 mm
Field-of-view	225 mm–240 mm	340 mm
Parallel imaging	None	Ipat factor = 2
Sampling matrix	128–160	256
Flip angle for readout	60^O^	90^O^/180^O^
Echo time	1.4 ms	T1-weighted: 29 ms
		T2-weighted STIR: 47 ms
Echo train length/shot	95 ms	T1-weighted: 70 ms
		T2-weighted STIR: 108 ms
Echo spacing	2.7 ms	T1-weighted: 5.8 ms
		T2-weighted STIR: 7.2 ms
Sampling bandwidth	1002 Hz/pixel	T1-weighted: 305 Hz/pixel
		T2-weighted STIR: 235 Hz/pixel
Magnetization preparation	Dual-inversion for dark blood	Dual-inversion for dark blood
	T2prep (T2_prep_ time = 60, 90 ms) for T2-weighting	T1-weighted: Chemical shift-selective RF for fat saturation; STIR: short-tau (TI = 170 ms) inversion
	Chemical shift-selective RF for fat saturation	
# of RR per trigger	T1-weighted: 1	T1-weighted: 1
	T2-weighted: 2	T2-weighted STIR: 2
Scan time/slice	T1-weighted: 4 × RR	T1-weighted: 9 (8 shots + 1 dummy rep) × RR
	T2-weighted: 8 × RR	T2-weighted STIR: 14 (13 shots + 1 dummy rep) × RR

Images were blindly reviewed by two independent readers, one radiologist (MPFB) and one cardiologist (AP), both with more than 5 years of experience interpreting cardiovascular imaging. Overall image quality, fat suppression and blood nulling were scored in a 5-point Likert scale as: 1- non-diagnostic; 2- poor; 3- fair; 4- good; 5- excellent. The quality of visualization of the right-ventricular (RV) free wall, left-ventricular (LV) free wall and septum were individually scored. Streaking and ghosting artifacts were noted, as well as signal dropout in the free wall of the left ventricle. The presence of incomplete blood suppression at the myocardial edge was also noted.

Statistical analyses were performed using R software (version 3.2.1, The R Foundation for Statistical Computing, Vienna, Austria). All analyses comparing radial bSSFP and Cartesian fast spin-echo images were done pair-wise based on the subject number, acquisition plane (short-axis vs. four-chamber), and image contrast (T1-weighted vs. T2-weighted). Ordinal Likert scores from both readers were averaged prior to analysis, and differences in radial bSSFP and Cartesian fast spin-echo scores were assessed using Wilcoxon signed-rank tests. Cohen’s kappa (κ) and Gwet’s AC1 were used to assess inter-reader agreement of scoring data. Artifact, signal dropout, and incomplete blood suppression were considered present if noted by at least one reader. These data were compared using McNemar tests. For each criterion scored, data were statistically analyzed in aggregate (including T1-weighted, T2-weighted, as well as short-axis and four-chamber images), and after stratification by contrast (T1-weighted and T2-weighted) and acquisition plane (short-axis and four-chamber). *P* values less than 0.05 were considered statistically significant.

## Results

**Fig. 1:**
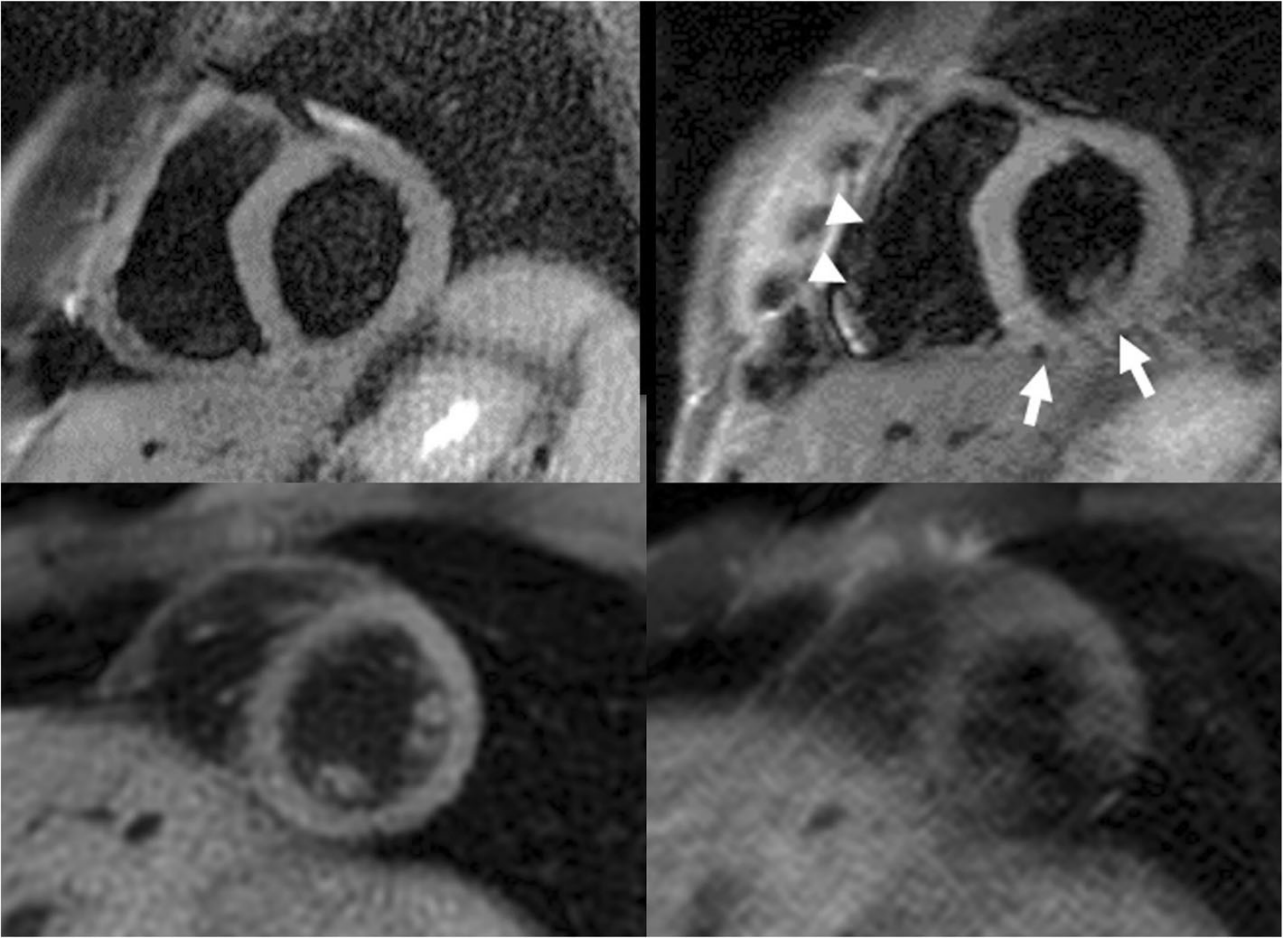
Comparison of short-axis dark blood radial bSSFP (4-shots, 140 views) with dark blood Cartesian fast spin-echo. Top row: Breath-hold scans. The ventricular myocardium is well shown with radial bSSFP (*left*), whereas with Cartesian fast spin-echo (readout bandwidth 305 Hz/pixel) (*right*) there are motion artifacts that obscure the inferior wall (*arrows*) of the left ventricle and free wall of the right ventricle (*arrowheads*). Bottom row: Free-breathing scans in a different subject. Radial bSSFP (*left*) shows good image quality with only mild blurring, whereas Cartesian fast spin-echo (readout bandwidth 977 Hz/pixel) (*right*) is non-diagnostic due to severe ghost artifacts and myocardial signal dropout

**Fig. 2:**
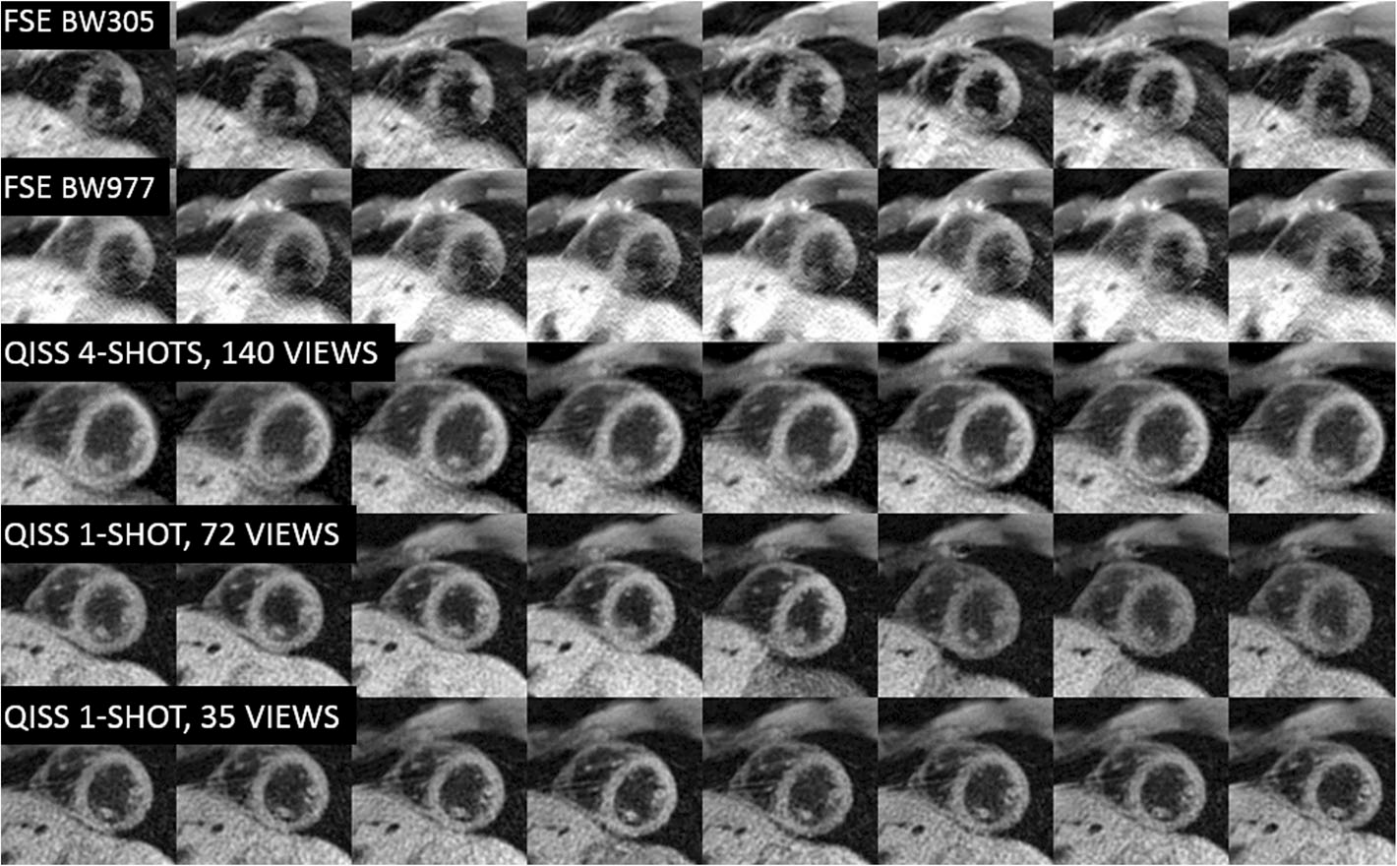
Montage of eight sequential images acquired in a healthy volunteer during free-breathing for five protocols. Single-shot radial QISS using 35 views shows the most consistent image quality and fewest motion-related artifacts. However, four-shot radial QISS with 140 views shows higher SNR. Both the single-shot 35-view and four-shot 140-view radial QISS scans have temporal resolution of 95 ms per shot, whereas the single-shot 72-view scan has a temporal resolution of 194 ms

In comparing radial bSSFP with Cartesian fast spin-echo, the most consistent difference was in the respective sensitivity to artifacts from cardiac or respiratory motion. Ghost artifacts from respiratory or cardiac motion sometimes obscured portions of the left ventricle in fast spin-echo images, but not with radial bSSFP (Fig. 1). The thin free wall of the right ventricle was routinely shown using radial bSSFP, but was inconsistently demonstrated with fast spin-echo. Similarly, fine trabeculations in the right ventricle were better shown with radial bSSFP. With free breathing, the fewest motion artifacts were seen using single-shot radial bSSFP with 35 views (Fig. 2).

Statistically significant differences in myocardial-chamber CNR, myocardial tSNR and sharpness of the LV endocardial-blood pool border were found across the five dark-blood protocols (*P* < 0.01) (Table 2). Under breath-hold conditions, arterial-to-blood CNR was largest with the low-bandwidth FSE protocol as compared to all other protocols (*P* < 0.05), while the 4-shot bSSFP protocol provided significantly larger CNR than the 1-shot bSSFP and high-bandwidth FSE protocols (*P* < 0.05). Temporal SNR measured during free-breathing was largest with the 4-shot bSSFP protocol; this value statistically differed from both 1-shot bSSFP protocols as well as the low-bandwidth FSE protocol (*P* < 0.05). Myocardial sharpness values during breath-holding were comparable with the 1-shot 35-view bSSFP, 4-shot bSSFP and low bandwidth FSE protocols, and lowest with the 1-shot 72-view bSSFP protocol. Under free-breathing conditions, myocardial sharpness was best with the 1-shot 35-view bSSFP protocol, which statistically differed with respect to all other protocols (*P* < 0.05) except for the low-bandwidth FSE protocol.

**Table 2:** Quantitative analysis of breath-hold and free-breathing scans in healthy volunteers

Measure	Protocol 1	Protocol 2	Protocol 3	Protocol 4	Protocol 5	ANOVA *P*-value
	bSSFP	bSSFP	bSSFP	FSE	FSE	
	1 shot 72 views	1 shot 35 views	4 shots	BW305	BW977	
CNR (breath-hold)	15.1 (4.3)^ab^	13.6 (5.4)^ab^	24.2 (7.2)^a^	46.7 (17.6)	24.5 (13.8)^a^	<0.001
tSNR (free-breathing)	7.0 (2.5)^abc^	9.3 (1.8)^b^	20.2 (5.2)	8.9 (3.0)^b^	12.9 (6.6)	<0.001
Sharpness (breath-hold)	0.30 (0.10)	0.43 (0.03)^d^	0.47 (0.09)^d^	0.47 (0.07)	0.38 (0.13)^a^	<0.01
Sharpness (free-breathing)	0.31 (0.10)^c^	0.49 (0.11)	0.30 (0.06)^c^	0.38 (0.11)	0.29 (0.08)^ac^	<0.01

Data presented as mean (standard deviation) across *n* = 6 subjects, except for protocol 2 where *n* = 5. *P* values for ANOVA and post-hoc analyses derived from *n* = 5 paired data

^a^ *P* < 0.05 versus protocol 4, FSE BW305

^b^ *P* < 0.05 versus protocol 3, bSSFP 4shots

^c^ *P* < 0.05 versus protocol 2, bSSFP 1shot 35views

^d^ *P* < 0.05 versus protocol 1, bSSFP 1shot 72views

*bSSFP* balanced steady-state free precession, *FSE* fast spin echo, *BW* receiver bandwidth, *ANOVA* repeated-measures analysis of variance, *CNR* contrast-to-noise ratio, *tSNR* temporal signal-to-noise ratio

For the retrospective clinical study, image quality scores are summarized in Table 3. Merging data across the two contrasts (T1-weighted and T2-weighted) and the two acquisition planes (short-axis and four-chamber) (*n* = 41 paired image sets), preference for radial bSSFP over standard-of-care Cartesian fast spin-echo was highly significant for overall image quality (3.9 ± 0.59 vs. 3.4 ± 0.74, *P* < 0.001), fat suppression (4.2 ± 0.4 vs. 3.5 ± 0.53, *P* < 0.001), RV free wall visibility (3.8 ± 0.75 vs.2.8 ± 0.88, *P* < 0.001), LV free wall visibility (4.3 ± 0.67 vs. 3.6 ± 0.88, *P* < 0.001) and septum visibility (4.5 ± 0.6 vs. 4.0 ± 0.74, *P* < 0.001). Inter-reader agreement data are summarized in Table 4. Aggregating scores from both contrast weightings, inter-reader agreement was generally moderate to substantial for all scoring criteria, except for fat suppression.

**Table 3:** Qualitative scoring data

			Image quality		Fat suppression		Blood nulling		RV wall visibility		LV wall visibility		Septum visibility	
Weighting	Slice Orientation	N	Radial bSSFP	Cart. FSE	Radial bSSFP	Cart. FSE	Radial bSSFP	Cart. FSE	Radial bSSFP	Cart. FSE	Radial bSSFP	Cart. FSE	Radial bSSFP	Cart. FSE
T1	Short-axis	13	4.2 (0.4)*	3.7 (0.5)	4.3 (0.3)^**^	3.7 (0.3)	4.3 (0.6)	4.3 (0.3)	4.1 (0.6)^**^	3.1 (0.8)	4.7 (0.3)*	3.9 (0.7)	4.7 (0.4)*	4.2 (0.5)
T1	Four-chamber	7	3.8 (0.3)*	3.0 (0.5)	3.8 (0.4)*	3.1 (0.8)	3.5 (0.6)	3.1 (0.7)	3.9 (0.3)*	2.6 (0.4)	4.1 (0.2)	3.9 (0.4)	4.3 (0.3)*	3.5 (0.4)
T1	Any	20	4.0 (0.4)^**^	3.4 (0.6)	4.2 (0.4)^***^	3.5 (0.6)	4.0 (0.7)	3.8 (0.7)	4.0 (0.5)^***^	2.9 (0.7)	4.5 (0.4)^**^	3.9 (0.6)	4.5 (0.4)^**^	4.0 (0.6)
T2	Short-axis	20	3.7 (0.7)*	3.2 (0.9)	4.2 (0.4)^***^	3.6 (0.5)	3.4 (0.7)^**^	4.0 (0.6)	3.7 (0.9)^**^	2.7 (1.0)	4.1 (0.8)^**^	3.3 (1.0)	4.4 (0.8)	4.0 (0.9)
T2	Four-chamber	1	3	4	4	4	3	3.5	3.5	3	4	4	4	4
T2	Any	21	3.7 (0.7)	3.3 (0.9)	4.2 (0.4)^***^	3.6 (0.5)	3.4 (0.7)^**^	4.0 (0.6)	3.7 (0.9)^**^	2.7 (1.0)	4.1 (0.8)^**^	3.4 (1.0)	4.4 (0.8)	4.0 (0.9)
all comparisons		41	3.9 (0.6)^**^	3.4 (0.7)	4.2 (0.4)^***^	3.5 (0.5)	3.7 (0.8)	3.9 (0.7)	3.8 (0.8)^***^	2.8 (0.9)	4.3 (0.7)^***^	3.6 (0.9)	4.5 (0.6)^***^	4.0 (0.7)

N denotes the number of pairwise comparisons

Values from both readers were averaged. Data presented as mean (standard deviation)

^*^ *P* < 0.05, ^**^ *P* < 0.01, ^***^ *P* < 0.001 versus Cartesian (Cart.) FSE protocol; Wilcoxon signed-rank test

**Table 4:** Inter-reader agreement

		Image quality		Fat suppression		Blood nulling		RV wall visibility		LV wall visibility		Septum visibility	
Weighting	Metric	Radial bSSFP	Cart. FSE	Radial bSSFP	Cart. FSE	Radial bSSFP	Cart. FSE	Radial bSSFP	Cart. FSE	Radial bSSFP	Cart. FSE	Radial bSSFP	Cart. FSE
T1 (*N* = 20)	κ	0.20	0.63^a^	0.26^a^	0.17	0.67^a^	0.48^a^	0.29^a^	0.56^a^	0.31	0.56^a^	0.25	0.46^a^
	AC1	0.50^a^	0.63^a^	−0.10	0.12	0.40^a^	0.47^a^	−0.05	0.36^a^	0.20^a^	0.36^a^	0.10	0.21
T2 (*N* = 21)	κ	0.66^a^	0.65^a^	0.07	0.05	0.63^a^	0.48^a^	0.71^a^	0.63^a^	0.59^a^	0.64^a^	0.50^a^	0.69^a^
	AC1	0.56^a^	0.43^a^	−0.15	0.35^a^	0.52^a^	0.28	0.31^a^	0.24	0.38^a^	0.24	0.42^a^	0.26
All weightings (*N* = 41)	κ	0.56^a^	0.65^a^	0.15^a^	0.12^a^	0.71^a^	0.49^a^	0.59^a^	0.61^a^	0.55^a^	0.63^a^	0.41^a^	0.61^a^
	AC1	0.56^a^	0.54^a^	−0.13	0.24^a^	0.50^a^	0.39^a^	0.21^a^	0.29^a^	0.46^a^	0.35^a^	0.44^a^	0.28^a^

N denotes the number of data points. *Cart.* Cartesian

^a^Denotes statistically significant agreement (*P* < 0.05)

**Fig. 3:**
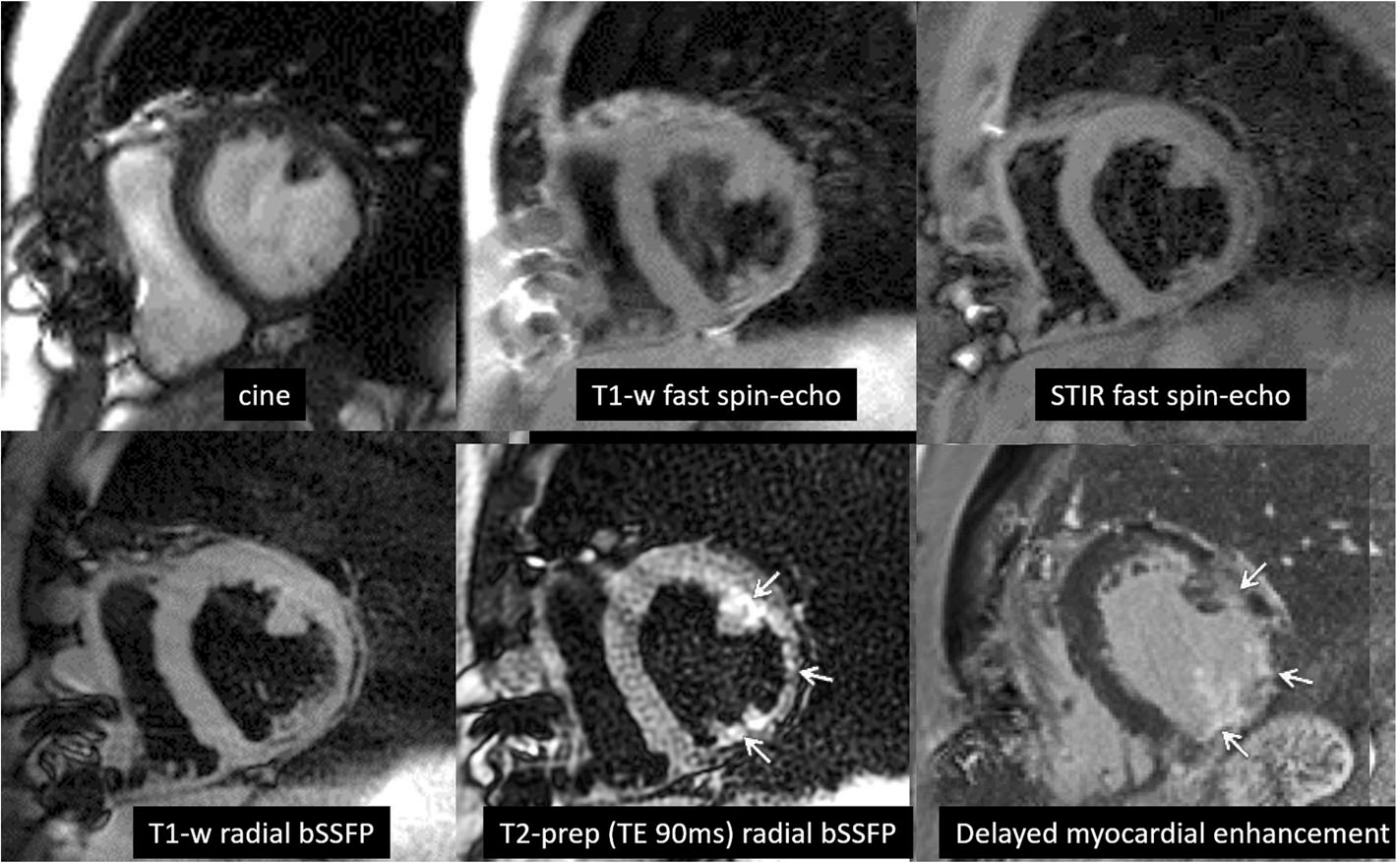
Patient with ischemic cardiomyopathy and history of coronary artery bypass grafts. Top row from *left* to *right*: systolic frame from cine bSSFP, T1-weighted fast spin-echo, T2-weighted STIR fast spin-echo. Bottom row from left to right: T1-weighted radial bSSFP, T2-weighted (T2_prep_ time = 90 ms) radial bSSFP, delayed myocardial enhancement using inversion recovery spoiled gradient-echo. The cine image shows inferolateral left ventricular wall thinning. The T1-weighted and T2-weighted fast spin-echo images show wall thinning but are otherwise unremarkable. In comparison with fast spin-echo, T1-weighted radial bSSFP appears less blurred. T2-weighted radial bSSFP shows a distinct subendocardial T2-signal abnormality corresponding to the region of delayed enhancement, which was not prospectively identified in the fast spin-echo images. (Low signal regions in the anterior chest wall are caused by sternal wires)

**Fig. 4:**
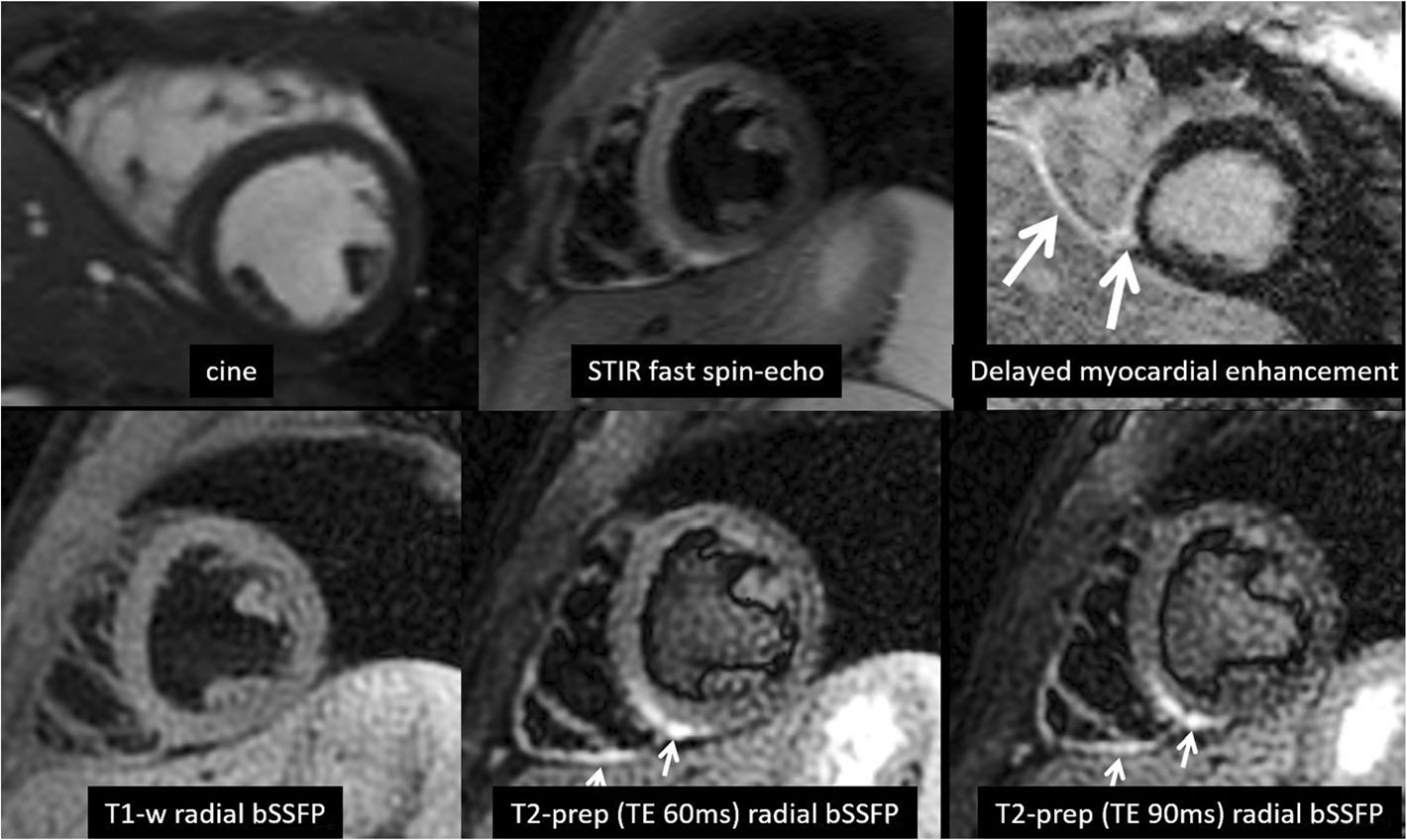
Patient presenting with chest pain and CMR findings consistent with myocarditis. Top row (from *left* to *right*): cine, T2-weighted STIR fast spin-echo, myocardial delayed enhancement using inversion recovery spoiled gradient-echo. Bottom row (from *left* to *right*): T1-weighted radial bSSFP, T2-weighted radial bSSFP with T2_prep_ time = 60 ms, T2-weighted radial bSSFP with T2_prep_ time = 90 ms. The T2-signal abnormality involving the inferior walls of the left and right ventricles is seen in the T2-weighted STIR fast spin-echo image, but is better delineated with T2-weighted radial bSSFP. Right ventricular trabeculations are also better delineated with the radial acquisitions. In this example, the blood pool was effectively nulled in the T2-weighted STIR fast spin-echo and T1-weighted radial bSSFP images, whereas the left ventricular blood pool has intermediate signal intensity in the T2-weighted radial bSSFP image due to suboptimal setting of the time delay following the dual-inversion preparation

For T1-weighted dark blood images (*n* = 20 paired image sets), preference for radial bSSFP over Cartesian fast spin-echo was highly significant for overall image quality (*P* < 0.01), fat suppression (*P* < 0.001), RV free wall visibility (*P* < 0.001), LV free wall visibility (*P* < 0.01), and septum visibility (*P* < 0.01). These differences remained significant for short-axis (*n* = 13) as well as four-chamber images (*n* = 7), with the exception of LV free wall visibility for four-chamber imaging. For T2-weighted images (*n* = 21 paired image sets), short-axis radial bSSFP images (*n* = 20) provided significantly improved overall image quality (*P* < 0.05), fat suppression (*P* < 0.001), RV free wall visibility (*P* < 0.01) and LV free wall visibility (*P* < 0.01). In two cases (Figs. 3 and 4), subtle T2-signal abnormalities were better shown using a T2-prepared radial bSSFP sequence than by the T2-weighted STIR fast spin-echo sequence. The degree of blood nulling was rated as better with T2-weighted STIR fast spin-echo than with T2-prepared radial bSSFP (*P* < 0.01). Examination of scores from both readers revealed that blood nulling was more frequently scored as “good” instead of “fair” (23 vs. 8 instances) with T2-weighted STIR fast spin-echo than with T2-prepared radial bSSFP (16 vs. 20 instances). Importantly, however, there were no instances in which blood suppression with either protocol was bad enough to merit a score of “non-diagnostic.”

The frequencies with which LV signal dropout, incomplete blood suppression at the myocardial edge, and either streaking or ghosting artifact were observed are provided in Table 5. Irrespective of slice orientation and imaging contrast, radial bSSFP provided fewer instances of LV myocardial signal dropout than Cartesian fast spin-echo (7/41 vs. 18/41, *P* < 0.05). For T1-weighed images, radial bSSFP more often demonstrated streak artifact than Cartesian fast spin-echo showed ghosting artifact (16/20 vs. 8/20, *P* < 0.05). However, the intensity of streaking artifact was mild and insufficient to interfere with image interpretation.

**Table 5:** Frequency of artifacts

		LV signal dropout		Incomplete blood suppression near myocardium		Streaking or ghosting artifact^a^	
Weighting	Slice Orientation	Radial bSSFP	Cartesian FSE	Radial bSSFP	Cartesian FSE	Radial bSSFP	Cartesian FSE
T1	short-axis	2/13	4/13	0/13	0/13	10/13	7/13
T1	four-chamber	1/7	0/7	1/7	5/7	6/7	1/7
T1	any	3/20	4/20	1/20	5/20	16/20*	8/20
T2	short-axis	4/20^**^	14/20	2/20	3/20	7/20	7/20
T2	four-chamber	0/1	0/1	0/1	1/1	1/1	0/1
T2	any	4/21^**^	14/21	2/21	4/21	8/21	7/21
all comparisons		7/41^*^	18/41	3/41	9/41	24/41	15/41

^a^Streaking artifact for radial imaging and ghosting artifact for Cartesian imaging

Data presented as number of instances/number of data sets

^*^ *P* < 0.05, ^**^ *P* < 0.01 versus Cartesian FSE protocol; McNemar test

## Discussion

Of the pulse sequences routinely used for CMR, the dark blood fast spin-echo technique is generally recognized to provide the least consistent image quality. In this study, we found that a dual-inversion, undersampled radial bSSFP technique outperformed standard-of-care Cartesian fast spin-echo technique at 1.5 Tesla, particularly with respect to image artifacts from cardiac and respiratory motion. Myocardial delineation was superior using the radial bSSFP approach. In particular, radial bSSFP was significantly better than Cartesian fast spin-echo for delineating the thin free wall of the right ventricle, which might be of particular benefit for detecting morphological abnormalities in cases of suspected right ventricular dysplasia.

In clinical practice, radial bSSFP has the potential to be advantageous in a variety of clinical scenarios, such as detecting small intra-cardiac masses or thrombi or subtle T2-signal abnormalities (e.g. from myocarditis or sarcoid). This latter benefit is supported anecdotally in two of our cases (Figs. 3 and 4) where small regions of T2-signal abnormality were better shown by T2-prepared radial bSSFP than by T2-weighted STIR fast spin-echo. However, further study with larger numbers of subjects and more optimal imaging parameters will be needed to validate the potential benefit of the T2-prepared radial bSSFP technique.

The use of radial imaging techniques dates back to the early history of MRI [12]. Although of great interest to researchers in the field of CMR, radial imaging techniques are seldom incorporated into standard-of-care CMR protocols (the main exception being for real-time, free-breathing cine acquisitions). A primary reason for this neglect may be the perception that radial imaging techniques are not robust. For instance, compared with Cartesian, radial images are more sensitive to artifacts from B_0_-dependent off-resonance effects, gradient-induced eddy currents, gradient timing errors, and non-uniform fat suppression. Moreover, given that standard-of-care imaging is almost exclusively performed with Cartesian techniques, lack of familiarity with radial imaging techniques makes it more difficult for physicians supervising CMR exams to optimize image quality or address radial-specific image artifacts such as streaking.

In contrast to these negative perceptions, our preliminary experience with a dark blood radial bSSFP technique shows several clinical advantages. For instance, radial k-space trajectories repeatedly sample the center of k-space, so that flow and bulk motion artifacts are minimized. Unlike Cartesian, where periodic bulk motion manifest as discrete ghost artifacts, in radial images motion manifests as blurring and streaking artifacts, which tend to be less obtrusive from a diagnostic standpoint.

Radial k-space trajectories are advantageous with respect to scan acceleration, since one can reduce the number of views without compromising spatial resolution so long as the intensity of radial streak artifacts is kept sufficiently low. In our study, streak artifacts were minimized by the use of equidistant azimuthal view angles, fat suppression, and strategic placement of spatial saturation pulses. This undersampling approach allowed us to reduce the echo train length of a single-shot dark blood acquisition to as little as 95 ms, while maintaining diagnostic image quality.

Because of the retrospective design of our patient study, we were limited to default standard-of-care imaging parameters. For instance, fast spin-echo used an 8 mm slice vs. 6 mm for radial bSSFP. In addition, only a low-bandwidth fast spin-echo protocol was available for patient studies. The low-bandwidth readout had the consequence of lengthening the inter-echo spacing, so that the protocol was less than half as efficient as four-shot radial bSSFP. By increasing the readout bandwidth for the volunteer study, the efficiency of the fast spin-echo protocol could be improved to nearly match that of the four-shot radial bSSFP protocol. However, even a high-bandwidth fast spin-echo protocol cannot come close to matching the efficiency of a single-shot, 35-view radial bSSFP protocol, which allows dark blood imaging of the entire heart to be easily completed within a single short breath-hold. Image quality for this highly-undersampled single-shot scan could be further improved through the use of iterative reconstruction techniques such as compressed sensing [13].

Off-resonance effects from B_0_ field inhomogeneity are a key concern with the radial bSSFP technique. Compared with Cartesian, radial k-space trajectories are more sensitive to artifacts from off-resonance effects. Moreover, bSSFP is much more sensitive to off-resonance effects than fast spin-echo. Consequently, it may seem counterintuitive to combine a radial k-space trajectory that is sensitive to off-resonance effects with a bSSFP readout that is also sensitive to off-resonance effects, especially since B_0_ homogeneity is disturbed at the heart-lung interfaces. Off-resonance effects with bSSFP, which manifest as periodic dark bands, were avoided by the use of a high bandwidth readout and short inter-echo spacing. Despite the high sampling bandwidth, SNR was adequate because the voxels used for dark blood imaging are relatively large.

Our MRI scanner automatically shims to the prescribed field-of-view. The small field-of-view used for radial bSSFP helps to ensure an optimal shim, so that off-resonance effects are minimized. So long as the small radial field-of-view is properly centered on the heart, large air-containing regions in the lungs and anterior to the chest wall are avoided during the shim procedure. Alternatively, one could use a large field-of-view and manually shim to the region of the heart, but doing so is cumbersome for technologists and more prone to operator error than the automatic shim procedure we used. It should be noted that the radial acquisition incorporates two-fold oversampling along the readout direction so that the use of a small field-of-view does not result in fold over artifact, unlike the case with a Cartesian k-space trajectory where fold over can occur along the phase-encoding direction.

The cardiac blood pool was generally well suppressed in short-axis views using both T1-weighted radial bSSFP and Cartesian fast spin-echo. Blood pool suppression was less consistent in the four-chamber view, presumably because slow-moving spins persist within the imaging slice, thus reducing the efficacy of the dual-inversion preparation. Blood pool nulling was also slightly worse (0.6 points lower on average) with T2-weighted radial bSSFP than with T2-weighted fast spin-echo, but was never rated as non-diagnostic. Fast spin-echo naturally tends to make through-plane flowing spins appear dark because excited spins flow out of the slice before refocusing (however, this property also makes FSE more prone than bSSFP to myocardial signal dropout from through-plane motion). Thus, fast spin-echo is probably less dependent than bSSFP on using an optimal time delay following the dual-inversion preparation in order to null the blood signal. Compared with T1-weighted scans, a longer time delay following the dual-inversion preparation will be needed for T2-weighted scans since gating to every second heartbeat allows for more T1 relaxation of blood pool spins. Better nulling of the blood pool with T2-weighted radial bSSFP could have been obtained by optimizing the choice of the time delay, which was not done in this study. The use of alternative techniques for suppressing the blood pool, such as DANTE pulses, might also prove helpful [14].

Our current study was performed entirely at 1.5 Tesla. However, we anticipate little difficulty in porting the dark blood radial bSSFP technique to 3 Tesla. Specific absorption rate should not be limiting given the modest excitation flip angle and short echo train length. However, further evaluation is needed with respect to off-resonance artifacts, which are likely to be substantially worse at 3 Tesla than at 1.5 Tesla. Finally, based on our experience with the optimized dark blood radial bSSFP technique, we anticipate that the use of a radial k-space trajectory might prove similarly advantageous for other cardiac applications such as cine, perfusion, and late gadolinium enhancement imaging [15].

## Conclusions

In conclusion, this study demonstrated that a radial bSSFP pulse sequence decreases motion artifacts and improves myocardial visibility and scan efficiency in comparison with standard-of-care Cartesian fast spin-echo for dark blood imaging of the heart.

## Abbreviations

bSSFP: balanced steady-state free precession
CMR: Cardiovascular magnetic resonance
QISS: Quiescent-interval slice-selective
STIR: Short tau inversion recovery
